# Decentralized Real-Time Anomaly Detection in Cyber-Physical Production Systems under Industry Constraints

**DOI:** 10.3390/s23094207

**Published:** 2023-04-23

**Authors:** Christian Goetz, Bernhard Humm

**Affiliations:** Hochschule Darmstadt— Department of Computer Science, University of Applied Sciences, 64295 Darmstadt, Germany

**Keywords:** anomaly detection, cyber-physical production systems, cyber-physical systems, deep learning, unsupervised learning

## Abstract

Anomaly detection is essential for realizing modern and secure cyber-physical production systems. By detecting anomalies, there is the possibility to recognize, react early, and in the best case, fix the anomaly to prevent the rise or the carryover of a failure throughout the entire manufacture. While current centralized methods demonstrate good detection abilities, they do not consider the limitations of industrial setups. To address all these constraints, in this study, we introduce an unsupervised, decentralized, and real-time process anomaly detection concept for cyber-physical production systems. We employ several 1D convolutional autoencoders in a sliding window approach to achieve adequate prediction performance and fulfill real-time requirements. To increase the flexibility and meet communication interface and processing constraints in typical cyber-physical production systems, we decentralize the execution of the anomaly detection into each separate cyber-physical system. The installation is fully automated, and no expert knowledge is needed to tackle data-driven limitations. The concept is evaluated in a real industrial cyber-physical production system. The test result confirms that the presented concept can be successfully applied to detect anomalies in all separate processes of each cyber-physical system. Therefore, the concept is promising for decentralized anomaly detection in cyber-physical production systems.

## 1. Introduction

**Figure 1:**
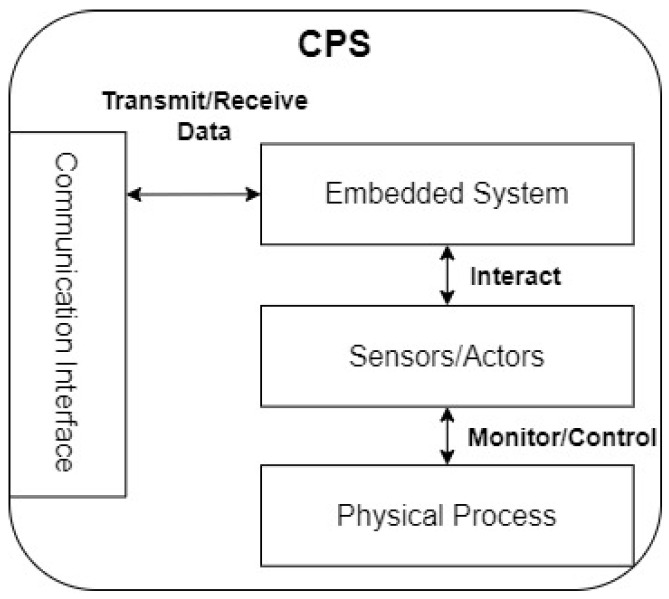
Abstract concept of a CPS [1].

Due to the rising complexity of modern processes in manufacturing, the application of cyber-physical systems (CPS) is increasing. A CPS can be described as a combination of an embedded system with sensors and actuators. The system interacts with these to monitor and control physical processes (Figure 1) [1]. Typically, the embedded system requires a communication interface to exchange data with other systems or a cloud. Many of these CPSs are networked to realize complex physical processes in the real world [2]. CPSs combine powerful information technology to monitor and control engineered systems [3].

Modern production systems, which include CPSs, are defined as cyber-physical production systems (CPPS) [4]. These systems are based on two main functionalities, advanced connectivity to ensure real-time data acquisition from the physical world and feedback from cyberspace. CPPSs break with the structure of the typical automation hierarchy to enable intelligent data management, real-time analytics, and enhanced computational capabilities. The control and field levels still exist to ensure the highest performance for critical loops, while the higher levels are more dynamic and decentralized [5].

**Figure 2:**
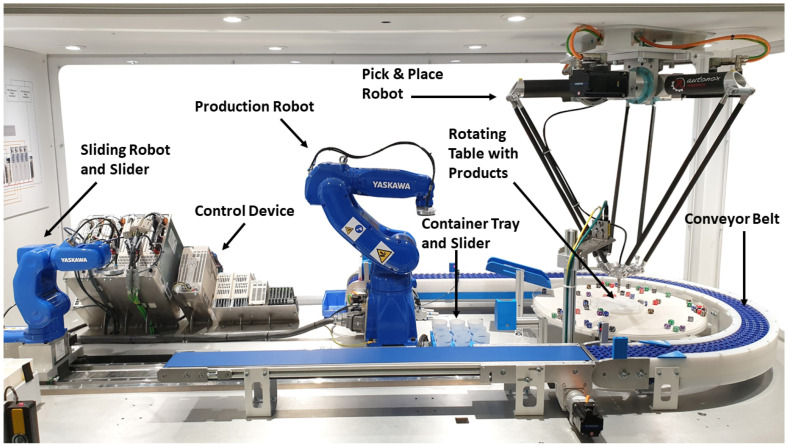
Rotary table dispenser system.

Such a CPPS can be seen in Figure 2. The rotary table dispenser system consists of different CPSs working together to realize several physical processes, e.g., transportation or pick-and-place operations. The overall process involves picking small items from a rotating table and putting them into several containers which are moving around the machine on conveyor belts. After the container is filled and reaches the end position, it gets picked up by the production robot and emptied back onto the rotating table. Thereafter, the container is put in a central location from which the sliding robot places it into the container tray. When the container tray is full, both sliders move to the left side of the system, and the sliding robot sets the container back on the conveyor belt. The described system acts as a simulation of a similar real industrial process and is used as a demonstration unit in Yaskawa. In total, there are nine CPSs, each combining a mechanical and an embedded system. Seven CPSs are based on servomotors and servo controllers. Two CPSs consists of an industrial robot with a robot controller. A central control unit collects data from the different CPSs and regulates the main production process. Additional computational units provide the opportunity to integrate higher functions, e.g., resource planning, production analysis, and process control handling.

In such a connected structure, even a single failure in one CPS can influence the entire production, resulting in a faulty product, a breakdown of the complete process, or a carryover of the failure through the whole system. Therefore, it is necessary to ensure an error-free operation to realize a secure and modern CPPS [6].

Anomalies can be taken as essential failure indicators, such as a rising vibration at a bearing of the rotating table or an unexpected torque increase on the motor of the conveyor belt. Anomaly detection (AD) in CPPSs refers to the identification of behavior that is not shown under the regular operations of the system. Consequently, by detecting anomalies, there is the possibility to recognize, react early, and in the best case, fix the anomaly to prevent the rise or the carryover of the failure throughout the entire manufacture [7].

Techniques for anomaly detection in CPPS can be distinguished into model-based [8] and data-driven approaches [9]. Model-based methods work based on precise and engineered models of the complete system. Creating such models over the complex structure of CPPS is time-consuming while simultaneously requiring deep expert knowledge. Data-driven approaches establish models only on collected data. Through the high amount of monitored and available data in CPPSs, these approaches are more appropriate for such systems, while additionally, no proper expert knowledge is needed [10]. Recent developments in machine learning and deep learning for anomaly detection have improved the detection performance on complex data sets [11].

By following the scheme to deliver all data from the control and field-level device to one CPS at a higher level to process, analyze, and detect anomalies, current centralized data-driven AD approaches in industrial CPPSs demonstrate better detection abilities than decentralized ones. While this is a significant advantage, it first requires a fully connected and high-performance unit for monitoring all integrated CPSs. Subsequently, adding such a unit increases costs and installation time. Additionally, a centralized concept creates a communication delay between the different stations to exchange the enormous amount of data produced in a CPPS. This can result in a delayed response after detecting an anomaly. Furthermore, it slows down the execution, evaluation, and detection of the anomalies in the individual CPS [12]. The structure of a CPPS is highly dynamic. Often single components, such as motors and sensors, are exchanged, replaced, or modified due to predictive or preventive maintenance. In a centralized approach, this results in a complete recreation of the AD due to the changed characteristics.

In contrast, a decentralized concept addresses these drawbacks by establishing the AD directly in each CPS. While this allows monitoring of the whole system by combining each separate AD, the need for a high-performance unit can be reduced, and the execution and response time can be increased. Furthermore, changes in a single CPS result in only the retraining of the associated AD. By establishing adequate prediction performances in each single CPS, comparable performance to a centralized AD can be reached.

The contribution of this paper is a novel unsupervised, decentralized, and real-time process anomaly detection concept for CPPS under industry constraints. We focus on industrial production processes and common constraints in CPPSs, including real-time requirements, asynchronous signals, prediction quality, configurable design, data-driven limitations, processing limitations, and communication interface constraints.

We employ several 1D convolutional autoencoders (1D-ConvAE) in a sliding window approach to achieve adequate prediction performance and fulfill real-time requirements. Current methods do not consider the limitations and constraints of industrial setups and mainly follow a centralized approach. By executing the installation process on an external, removable device, we increase the flexibility of our concept while considering processing limitations. To meet communication interface and processing constraints in typical CPPSs, we decentralize the execution of the AD into each separate CPS. The installation is fully automated to tackle data-driven limitations. Thereby, no expert knowledge about explicit anomalies is needed. Adjustments to the data collection routine were made to optimize the external sampling procedure and improve the installation process.

This paper is structured as follows. Section 2 summarizes related work about anomaly detection for industrial CPS and CPPS. The problem statement is specified in Section 3. Section 4 presents a concept for fast and decentralized unsupervised anomaly detection in CPPS. Information about a prototypical implementation is provided in Section 5. In Section 6, the evaluation of the approach is presented based on an industrial setup. Finally, a conclusion and an outlook for future work are given in Section 7.

## 2. Related Work

Surveys on anomaly detection techniques can be found in [13,14,15]. More industrial-related AD methods are described in [16,17]. Overall, these techniques can be differentiated into model-based and data-driven approaches. Model-based techniques detect anomalies by manually creating precise models about the underlying system. This requires a deep prior knowledge of the individual CPPS. While data-driven approaches are also based on models, those models are generated automatically from data and not manually by domain experts. Furthermore, data-driven approaches can be split into supervised and unsupervised techniques. Anomalous data in CPPS is associated with the undefined behavior of the system. Creating such anomalous data can be hazardous for the CPPS itself, while defining all possible anomalies in advance is nearly impossible. Based on the points mentioned above, we focus on unsupervised data-based methods.

Common approaches in unsupervised data-based AD are one-class classification methods, such as deep one-class networks [18] and one-class support vector machine [19,20]. While multi-class classification techniques typically require labeled datasets, these approaches focus on the normal samples by learning a discriminative hyperplane surrounding them. Other frequently used techniques are unsupervised clustering methods such as Gaussian Mixture Models [21], k-nearest neighbor methods [22], or random isolation forests [23]. These models can identify anomalies by building a detailed representation of the normal data. While the resulting models are generally lightweight and computationally fast, they lack performance when processing high-dimensional data.

Deep learning methods for AD have recently improved the state of the art in detection performance on complex and large datasets [24]. The standard techniques in this field are generative adversarial networks (GAN). GANs consist of a generator combined with a discriminator as the base structure. By teaching the discriminator to distinguish between real and fake samples while the generator tries to generate new data based on the input, GANs can detect anomalies even in large multivariate data streams. Concepts of GANs differ mainly in the models used as the base structure, such as long-short-term-memory (LSTM) recurrent neural networks (RNN) [25], two-dimensional convolutional autoencoder [26], and one-dimensional convolutional autoencoder [27]. While the described approaches achieve good outcomes, they result in highly complex and large models that cannot be applied to a CPS with limited computational resources, which is a common industry constraint.

Reconstruction-based methods in AD combine techniques that rely on the assumption that a model trained only on normal data cannot reconstruct abnormal or unseen data. Typical techniques of these fields are PCA methods [28] or sparse representations [29]. A widely used approach for reconstruction-based anomaly detection in CPS is using autoencoders [30,31] or variants thereof [32,33]. By learning the latent features of the input data, autoencoders can reconstruct their input as output. While these models can be applied to analyze the spatial characteristics of the input data, they miss considering the temporal dependencies, which are necessary indicators for anomalies in the time series data of industrial CPS.

While convolutional neural networks (CNN) were initially developed for solving image classification tasks, they can also be successfully applied for AD in time series data of a CPS through the ability to extract temporal dependencies [34]. Several industrial applications of CNNs in CPS, such as fault detection in motors [35], AD in wheelset bearings [22], and rolling bearings [36], can be found. Additionally, ref. [37] pointed out that CNNs have lower parameters than other network structures while performing comparably or better, resulting in reduced complexity, needed storage capacity, and computing power [38].

Convolutional autoencoders (ConvAE) combine the ability to detect temporal anomalies with the help of convolutions and spatial anomalies by the autoencoder structure while being also resource-efficient. This results in ideal models for AD in multivariate time series data [39,40,41]. Using a 2D variational ConvAE, the authors in [42] detect anomalies from unseen abnormal patterns in industrial robots. In [43], a ConvAE based on channel-wise reconstruction in combination with a local outlier factor is used to detect anomalies in automobile sensors.

Several approaches for decentralized AD can be found [44,45,46]. In [12], different decentralized AD techniques are analyzed and compared in complexity and performance. A decentralized approach for real-time AD in transportation networks is introduced in [47]. The authors of [48] presented spatial anomaly detection in sensor networks using neighborhood information. While these are promising approaches for decentralized AD, no work considers all the different industrial constraints simultaneously, which is important for integration into a CPPS.

Different automated frameworks for anomaly detection can be found. In [49], a framework for automatic time series anomaly detection is introduced. The study focuses on large-scale time series data in a centralized AD approach, which cannot be applied to a CPPS with limited resources. The authors of [50] introduce an unsupervised framework for anomaly detection in CPS. Furthermore, ref. [51] presents a high-performance unsupervised anomaly detection for CPS networks. Both approaches are developed for CPS, but mainly focus on adversarial attacks and not on the process of the CPS and, respectively, of the CPPS.

In our previous work [52], we introduced an unsupervised anomaly detection concept for CPSs under industry constraints while focusing on repetitive tasks with a fixed duration for a single CPS. In this contribution, we improved the concept for CPPS with multiple CPSs, while still considering all industrial constraints. We adapted the technology to a sliding window approach to simultaneously handle processes with variable durations and meet real-time instead of near-time requirements.

In summary, there are several approaches for centralized and decentralized data-driven unsupervised anomaly detection. Only a few are evaluated in real CPSs, and even fewer are applied to real production data of a CPPS. Overall, no work considers all the different industrial limitations of a CPPS while following a decentralized and fast approach to realize anomaly detection in industrial production data.

In this work, we propose a concept that addresses all the requirements that must be considered to realize a usable decentralized, real-time anomaly detection in CPPS under industrial constraints. Our contribution in this paper is summarized as follows. We employ several 1D-ConvAEs for unsupervised anomaly detection in a CPPS to monitor the different processes. We introduce a novel concept to decentralize the different models in each single CPS of the CPPS by splitting the installation and execution of the anomaly detection to meet industrial requirements. While the concept is fully automated, no expert knowledge about explicit known anomalies is needed to meet the defined requirements.

## 3. Problem Statement

This article aims at a decentralized concept for real-time unsupervised anomaly detection for production processes under industrial constraints. The problem statement can be described by the different industrial requirements that must be considered to implement such a concept. Several conditions are adapted and extended from [52].

1. **Anomaly detection:** An anomaly detection for a CPPS, such as an industrial production system, shall be performed. The CPPS consists of multiple CPSs producing multivariate time series data over variable process lengths, for example, the sliding robot from the CPPS in Figure 2, combining a robot with several axes and a robotic controller to move containers on a conveyor belt.
2. **Real-time:** To cover all different kinds of anomalies and react even in time-critical scenarios, such as detecting collisions in the production system, the result and reaction of the anomaly detection should be available as quickly as possible. Therefore, the execution of the anomaly should be performed during production, and the results must be immediately provided after new data from sensors and actors are available, e.g., a few milliseconds after the data is received.
3. **Prediction quality:** For an AD application in an industrial environment, adequate prediction performance is required. This depends on the different use cases for which the anomaly detection is applied, e.g., an F1 score of 0.95 or better for each CPS in the CPPS.
4. **Configurable:** To apply AD on different CPPSs in different applications, the anomaly detection should be adaptable to various CPSs and use cases. The possibility of using the technique for varied time series data with different variable types and diverse time lengths should be given, for instance, robots or transportation systems with features such as torque, position, and speed.
5. **Data-driven:** As mentioned before, manually creating models is time-consuming and requires deep expert knowledge. Simultaneously recording anomalous data from CPPS can be dangerous for the system itself. Therefore, the AD should only be trained with regular production data and without expert knowledge.
6. **Feasible:** The AD should be compatible with current technological standards in industrial environments to realize a generalist integration for various scenarios. This includes constraints and limitations of commonly used CPPSs in production settings:
  (a) Process limitations, due to the design of CPSs in industry, that are unable to execute process-intensive tasks in parallel to control and monitor the physical process, e.g., limited available RAM and processing power.
  (b) Communication interface constraints of commonly available CPSs in industry, e.g., OPC UA Communication, to transfer the high amount of production data at a sample rate of 2 ms during the sampling process to a database.

## 4. A Concept for a Fast, Decentralized, and Unsupervised Anomaly Detection in CPPSs

### 4.1. Overview

**Figure 3:**
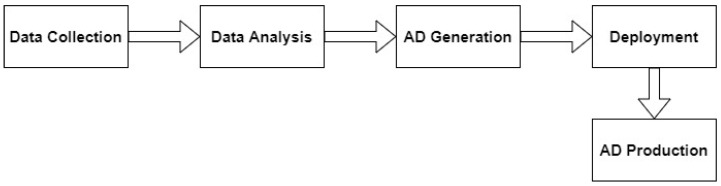
Sequence of steps performed in the concept.

**Figure 4:**
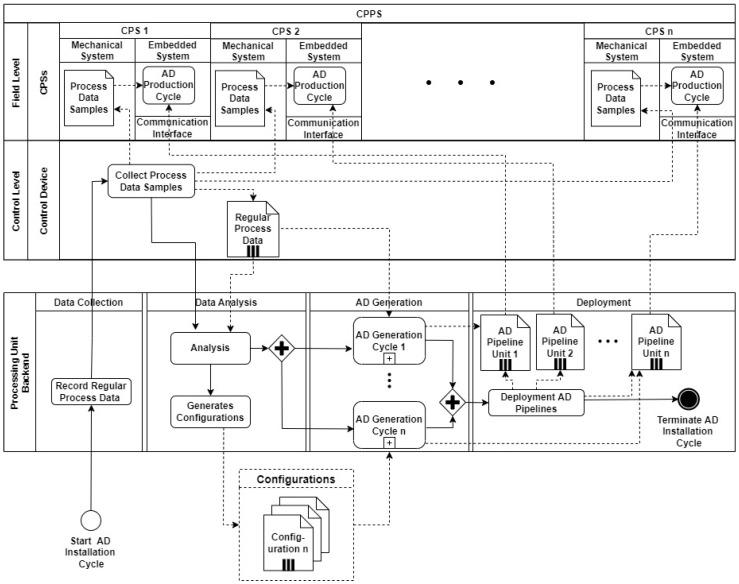
Overview of AD Installation Cycle.

**Figure 5:**
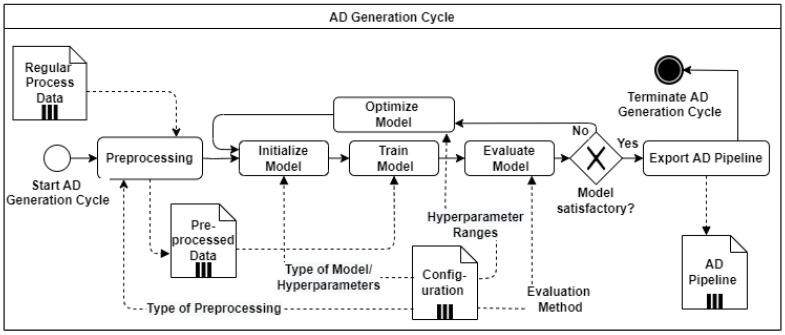
Overview of AD Generation Cycle.

**Figure 6:**
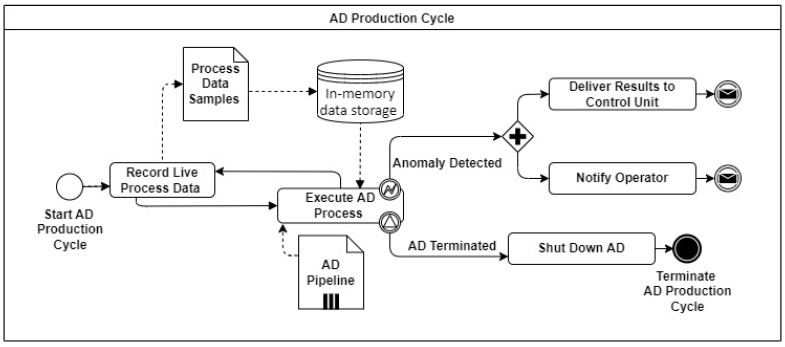
Overview of AD Production Cycle.

This section describes a fast and decentralized process anomaly detection concept based on several 1D-ConvAEs, which fulfills the requirements specified in the problem statement. Figure 3 shows the sequence of the different steps that are carried out. The concept consists of one AD Installation Cycle, which triggers the creation of several anomaly detection pipelines (AD pipelines) through the parallel execution of AD Generation Cycles, as shown in Figure 4. A detailed description of the AD Generation Cycle can be found in Section 4.3 and in Figure 5. The number of different AD Pipelines depends on the number of included CPSs in the CPPS. In the AD Production Cycles, located in every CPS in Figure 4, each pipeline is directly implemented and executed as part of the CPS. Explanations about the AD Production Cycle can be found in Section 4.4 and in Figure 6. The processing unit backend, an external device that can be removed after the installation process is finished, performs all heavy processing tasks of the AD Installation Cycle to meet the previously explained industrial constraints of the CPPS. The concept is developed to be executed automatically, enabling AD implementation without deep expert knowledge. In addition, a direct explanation of the individual components of the diagrams can be found in Appendix A.

### 4.2. AD Installation

The AD Installation Cycle consists of four parts: data collection, data analysis, AD generation, and deployment (see Figure 4).

**Data collection:** The operator triggers the data collection at the processing unit backend to record regular process data. Process data samples, single packages of time series data from the individual CPS, are collected at a high sample rate and sent to the control device. Over a defined period of time, the individual data of the various CPSs are recorded and then combined. The resulting package, named regular process data, is then sent to the processing unit backend. This procedure is required to meet the communication interface limitations in the installation process and enable the use of the high sample rates at the AD Production Cycles directly in the CPSs. The data packages are saved inside the processing unit backend until a specified number of records is reached. Regular process data consist of different features like position, torque, and speed sampled in the form of time series data from the various CPSs. This data can be defined as multiple data streams containing the features of the physical process recorded by the different sensors and actors.

**Data Analysis:** Depending on the diverse CPSs, different features with different ranges are provided. In the analysis step, unnecessary features are automatically removed, and configuration files are accordingly generated. Each configuration file contains the necessary information for the following AD generation cycle, e.g., feature ranges, types, and default hyperparameters. The operator can manually tune this information, or the default values can be used.

**AD Generation:** In the installation step for each included CPS, an AD Generation Cycle (Figure 5) is triggered. The different AD Generation Cycles can be executed in parallel to speed up the installation process. A detailed description of the AD generation cycle can be found in Section 4.3.

**Deployment:** After the generation of the AD pipelines, each pipeline is exported and deployed to the separate CPS. This terminates the AD Installation Cycle.

### 4.3. AD Generation Cycle

The AD Generation Cycle consists of preprocessing, model initialization, training, evaluation, optimization, and export, as shown as a BPMN diagram in Figure 5. First, the provided regular process data, the combined collected data samples of all CPS, are preprocessed with the information received from the configuration files. This transforms the data, which consist of different ranges and units, into an equal numerical range. The type of the desired preprocessor is defined in the configuration file. This enhances a configurable setup, which can handle various variables with different units and ranges. Next, the model is initialized, trained, evaluated, and optimized. Additional hyperparameters set in the configuration file are, e.g., the number of layers, filters per layer, used loss function, and type of optimizer. After initialization, the model is trained on the preprocessed data. The method specified in the configuration file is used to evaluate the model. In the optimization step, the hyperparameters are changed, influenced by the defined ranges and tuning parameters. The search algorithm declared in the configuration file searches over a generated search space for the best possible parameters. These steps are executed iteratively until the specified reconstruction performance (e.g., the desired MAE Value) is reached. After the tuning is finished, the AD pipeline, a combination of preprocessor and model, is exported to the deployment step. This terminates the AD generation cycle.

### 4.4. AD Production Cycle

After the AD Generation Cycle is finished and the AD pipeline is deployed in the CPS, the AD Production Cycle, shown as a BPMN diagram in Figure 6, starts. Process data samples, single packages of time series data from the CPS, are collected at a high sample rate and stored in an in-memory data storage. When the required amount of data packages to execute the AD process step is reached, the data are preprocessed and evaluated by the AD pipeline. After the execution, the previously collected data in the in-memory data storage will be released to limit the needed memory capacity. The AD process step will be executed again immediately after enough data is available. In case of an anomaly, the detection can be delivered to the control unit, or the operator can be directly notified. Additionally, the AD process can be terminated, and therefore the AD Production Cycle.

### 4.5. Sliding Window Convolutional Autoencoder

**Figure 7:**
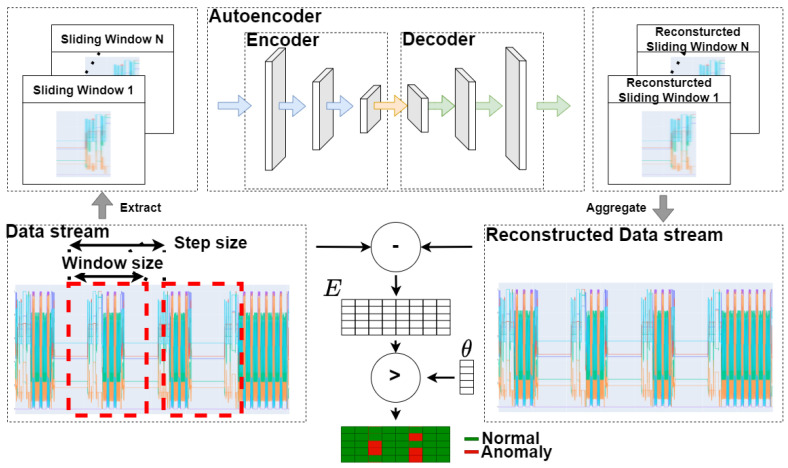
Convolutional autoencoder.

To achieve adequate prediction performance and meet real-time requirements, we choose a sliding-window-based 1D-ConvAE as the model type (see Figure 7). Autoencoders are reconstruction-based neural networks that reconstruct their input as output. By only learning the reconstruction of the regular pattern, every datum consisting of unseen, abnormal patterns cannot be correctly reconstructed, which will result in a higher reconstruction error. To gain adequate prediction performance and meet the processing limitations, 1D convolutional layers are used. Adding these layers to the autoencoder allows the model to learn spatially invariant features and capture spatially local correlations from the data. This means it can recognize patterns of high-dimensional data without requiring feature engineering. At the same time, the required parameters and the computational complexity of a 1D convolutional layer are significantly lower than the comparable 2D convolutional layers. The 1D-ConvAE can be trained without expert knowledge or explicitly known anomalies, only with regular process data. This fulfills the requirement 5, *data driven*. A detailed comparison with other methods can be found in Appendix B.

### 4.6. Anomaly Detection

Regular process data can be defined as a data stream containing several time series of sampled features $F=(f_{1},f_{2},...,f_{n})$, where n defines the number of different features such as position, speed, and torque from the various sensors of the mechanical system. The data stream is split into several windows depending on the chosen window size m and step size s. Each window consists of several time series equal to n different features in the data stream over a time period corresponding to the window size m. These generated sliding windows act as the input to the model. The output of the model is each separated reconstructed sliding window. With the help of an aggregation function (e.g., arithmetic mean), the reconstructed sliding windows can be merged into a reconstructed data stream. To calculate the reconstruction error matrix *E*, the reconstructed error $e_{i_{t}}$ of each feature $f_{i}$ at every time step t can be calculated as the Absolute-Error (AE) $e_{f_{i_{t}}}=|f_{i,t}-{\hat{f}}_{i,t}|\left(1\leq i\leq n\right)$ between the input and output. This results in a matrix *E* representing each feature at each time step as a value of the differentiation between the input and reconstructed data stream. Threshold values must be defined to evaluate which value a reconstruction error indicates if an anomaly is detected. We employ the following method for automatically computing and tuning the threshold values. After training the model, the described method re-evaluates all training data. This results in an error matrix over the whole training data stream. The maximum reconstruction error of each feature is taken from this matrix to construct a threshold vector θ. This vector can be adapted when the model is integrated directly into the CPS by automatically tuning the values in the live testing stage. In the AD production cycle, after enough high sample data are collected in the in-memory storage, each column of the reconstruction matrix *E* is evaluated with the threshold vector θ. The number of collected data samples can be flexibly chosen but must be at least twice as large as the window size to allow the reconstruction concept to be applied. Suppose a value of $e_{f_{i_{t}}}$, where *i* is the considered feature of the total features and *n* exceeds the associated threshold value of this feature $\theta_{f_{i}}\left(1\leq i\leq n\right)$. In that case, the data point in the input data stream is declared anomalous. Therefore, anomalies in the input data stream can be detected by applying the threshold vector θ to each timestep *t* of the reconstruction matrix *E*.

## 5. Prototype Implementation

The concept has been implemented prototypically. As programming language, Python 3.9 is used. A MongoDB https://www.mongodb.com/ (accessed on 12 February 2023) is established on the processing unit backend to save and export the regular process data. As a preprocessor, a MinMaxScaler was generated. The model is implemented using the Keras library https://keras.io/ (accessed on 12 February 2023), running on top of Tensorflow https://www.tensorflow.org/ (accessed on 12 February 2023) [53]. For hyperparameter tuning, the python library Ray Tune https://ray.io/ (accessed on 12 February 2023) [54] is used. Finally, a tracking server based on the library Mlflow https://mlflow.org/ (accessed on 12 February 2023) [55] was established to track the training results. The communication between the motion controller and the processing unit backend was realized through an OPC UA server–client model based on publish–subscribe routines. Several function blocks for buffering the high sample process data from the CPS at the motion controller were developed to establish this concept. This enables an intelligent communication pattern, where only minor changes on the motion controller must be performed to allow the described data exchange. The configuration files are written in YAML and can be accessed and changed by the operator. For each CPS, a separate configuration file is created. These files are also tracked to enable a traceable process at a later stage. The preprocessor and model integrations are developed as interfaces to satisfy the configurable requirement. Therefore, various considered models and preprocessors can be implemented as long as they follow the abstract class structure, making it easy to exchange, adapt, or evolve the described technique. To visualize the detection results and allow the user to interact with the system, a dashboard for bi-directional communication between the CPPS and the operator was implemented.

## 6. Evaluation

### 6.1. Experimental Setup

The rotary table dispenser system shown in Figure 2 was used to evaluate the decentralized concept. The CPPS consists of different CPSs and a control unit working together to realize several processes, e.g., transport and pick-and-place operations. The overall process involves picking small items from a rotating table and putting them into several containers which are moving on conveyor belts around the machine. During the process, the different time series data of each CPS is collected in the motion controller. Several buffers are written in the motion controller to adapt the high sample rate of 2 ms of each CPS to the minimal data exchange cycle time of 50ms at the OPC UA server. After one buffer is filled, the data package is sent to the OPC UA Server running on the processing unit backend, a pc type NUC8i5BEK. The collected data are saved in the established database after each import cycle. In total, 33 different time series over a period of 6 min were recorded. Based on the high sample rate, each time series consists of around 176,000 samples, resulting in approximately 5,808,000 data points as training data.

### 6.2. Data Recording

Realistic fault data were generated by forcing different anomalies into the normal process to evaluate the performance of the used models. The resulting deviations from the normal process were manually classified as anomalous areas in the resulting data stream to rate the performance. Five error cases were defined, and at least one error case was generated for each CPS. Additionally, long-term tests, including several complete processes without anomalies, were carried out to control the resulting models in the normal industrial setup.

1. **Friction:** To simulate friction, which can result from abrasion of used mechanical components, delayed maintenance, or broken parts, external forces were applied to the mechanical systems of the different CPSs, e.g., against the rotation direction of the conveyor belt or the movement of the linear sliders. This results in increased torque values at the applied CPS.
2. **Vibration:** Undefined vibration, which can be caused by broken bearings or loose attachments, was applied to the mechanical system of the CPS. The simulation was done by manually applying shocks to the rotating table.
3. **Defect components:** Another industry-related anomaly can be caused by defect components in the production process, such as a broken container. To examine this type of anomaly, different containers were manipulated in such a way that they could not be picked by the robots anymore, resulting in an undefined status of the whole production line.
4. **Incorrect process:** In addition, external manipulations can influence industrial production lines. These injections in the normal process can result in some undefined behavior of the system, which can cause damage to the products or the system itself. To simulate this kind of anomaly, the placement of the containers on the belt was changed in the running process. Therefore, the real positions differ from the fixed pre-defined positions in the machine scope.
5. **Collision:** Due to external influences or process errors, even in modern industrial systems, collisions may occur. The system typically detects heavy collisions, whereas smaller collisions resulting in damaged products or fragile components are mostly not recognized by the internal system. This can be, for example, a collision with an obstacle in the moving path of the linear sliders or a displaced product on the conveyor.

### 6.3. Model Configuration

The sampled data from the regular process was used to train the model. A MinMax Scaler was chosen to preprocess the data by scaling the time series between zero and one. An Adam optimizer was used, and the loss function was set to MAE. The default hyperparameter tuning results in 80 different decoder and encoder structures for each CPS. The best model was automatically picked by evaluating the number of parameters and the resulting loss value. Detailed information about the different considered parameters can be found in Table 1. The focus was on realizing small and efficient model architectures to meet the computational limitations (Section 3, point 6). Therefore, shallow structures with a limited amount of parameters were preferred. By comparing all achieved loss values and the resulting model structures, the smallest structure that achieved a low loss value and, thus, a good reconstruction capability was automatically selected. Due to the unsupervised setup, no anomalous data are available in the training process. Therefore, an immediate evaluation of the detection performance is not possible; consequently, only the reconstruction capability can be taken as an additional selection criterion in this process. Additional discussion on the selection process can be found in future work. As activation function, the rectified linear unit was chosen. Dropout layers were applied as regularization between the convolutional layers, and max pooling layers were used to reduce the dimensionality. Different step sizes in the training process were tested. The best results were reached with a step size of one.

### 6.4. Experimental Results

This section validates the described concept applied in the experimental setup against the requirements defined in the problem statement.

**Figure 8:**
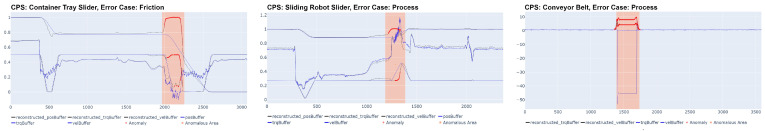
Anomalous Samples.

1. **Anomaly detection:**Figure 8 shows some of the forced anomalies in the experimental setup, illustrating the detection performance of the generated models. In the pictures, the detected anomalies are marked with red points, while the pre-defined anomalous areas are indicated by the red background color of the figure. Combined with the results in Table 2, this confirms that the different models can be successfully applied to detect anomalies in the CPSs.
2. **Real-time:** The evaluated sliding window sizes from the hyperparameter tuning were between 32–64, resulting in comparably small windows. To ensure a fast detection in the real process, each generated sliding window was treated as a data stream and evaluated immediately. With a sample rate of 2 ms, the overall time to collect one window as input data for the model is between 64 and 128 ms. The average execution time per reconstruction and verification for anomalies was around 34 ms, with a maximum of 49 ms and a minimum of 22 ms. Therefore, anomaly detection can be carried out with a maximum delay of 177 ms at our setup, which allows an immediate reaction of the system on detected anomalies.
3. **Prediction quality:** The F1 Score is used to evaluate the model performance. The detailed performance for each CPS is shown in Table 2. To calculate the F1 Score, the manually forced anomalies were classified as anomalous areas. If an anomaly in a window was detected, the used window was assigned as anomalous and evaluated against the area. By reaching high F1 Scores above 0.95, adequate prediction performances for every single CPS are realized. This confirms that the automatically created models for each CPS can reliably detect anomalies in the given CPPS.
4. **Configurable:** The described concept and resulting anomaly detection can be configurated for various applications. Only minor changes must be made to the motion controller to enable the sampling process. The automatically generated configuration files can be manually changed, or the default values can be used.
5. **Data-driven:** The models are trained only with the regular process data. Therefore, no anomalous data or feature engineering is needed. No values are added or changed. All removed features are automatically declared. Only the data from the sensors and actors of the CPSs are used. The model is created in an automated way by the configuration file without the need for expert knowledge.
6. **Feasible:** The method utilized standard communication technologies of common industrial setups. By outsourcing the process-intensive tasks to the processing unit backend, the concept enables the application of anomaly detection for the CPPS, even with the processing limitation and constraints of each CPS. In our experimental setup, the simulated process reaches a maximum consumption of 350MB while not exceeding a maximum of 12% CPU load.

Based on the experimental results, the introduced novel concept fulfills all the defined industrial requirements of the problem statement in Section 3.

## 7. Conclusions and Future Work

This paper presents a fast and decentralized anomaly detection concept for CPPS under industry constraints. The concept is configurable and feasible to apply anomaly detection in different use cases under the limitations of commonly used CPPSs in industrial environments. Due to the decentralization, no additional computational units must be integrated. The generated models allow a fast and performant integration. The anomaly detection is executed, and evaluations are carried out immediately during production. The model is generated and tuned in a fully automated fashion. No expert knowledge about anomalous data is needed. Overall, the experiments show that each model achieves stable and accurate results. This presents a promising approach for decentralized and fast anomaly detection in CPPSs under industry constraints.

However, despite the apparent success of the concept, there are several directions for future research. In this work, the concept was only tested in a single CPPS with a limited amount of CPSs. Therefore, more studies with different models, more CPSs, and under different scenarios will be performed in future work. Secondly, the models are only evaluated against the defined simulated computational resources and data storage limitations of the used CPSs. This is mainly caused by the integration limitations of the available CPSs. To integrate the models, adaptions to the hardware and software of the CPSs must be carried out in the future. Additionally, several anomalies which can emerge in a CPPS cannot be detected, e.g., process anomalies such as changing the overall process to fewer containers as in the learning process. This forces the CPPS not into an undefined state, although the actual process differs from the learned process. Therefore, another research direction in the future is to adapt the concept even to detect this kind of anomaly. Furthermore, the selection of the model is only based on two parameters, the achieved loss value and the resulting model structure. Despite the good results obtained in the tests with the defined anomalies, this method cannot guarantee the selection of the best model. Further approaches and concepts for a better evaluation of the models and a guaranteed choice of the best model must be found.

Finally, up to now, the output of the anomaly detection is the identification of the anomaly, defined by the time and feature, in the data stream. Adding more information may be helpful to increase the accuracy of the AD for the operator. Ways to gather and provide this additional context information will be evaluated and investigated.

## Figures and Tables

**Table 1:** Summary table of all parameters taken in the process of automatically selecting the models.

Model Parameter	Range	Definition
Number of Layers	[4, 8]	Total number of layers used in the model.
Number of Filters in the first Layer	[32, 128]	The number of filters used in the first layer of the model. To realize the dimensionality reduction, the inner layers have fewer filters. (In the automated concept, half of the previous layer).
Window size	[32, 128]	Number of time steps of the sliding window.
Step size	[1, 64]	The length of the sequence shifted between the individual windows.
Patience	[1, 10]	Number of epochs with no improvement after which training will be stopped.
Total number of parameters	[12, 642, 208, 614]	Total number of parameters of the resulting model.
Mean absolute error	[0.002, 0.3]	Achieved mean absolute error between input and output at the end of training.

**Table 2:** Performance Evaluation.

Unit	TP	TN	FP	FN	Precision	Recall	F1-Score
CB	232	3938	7	10	0.0.9707	0.958	0.964
RT	193	3199	9	8	0.955	0.960	0.957
SR	22	3002	2	0	0.916	1	0.956
PR	22	3002	1	1	0.956	0.956	0.956
P&P S	484	2965	21	20	0.958	0.960	0.959
P&P U	484	2967	29	15	0.943	0.969	0.956
P&P L	484	2964	27	22	0.947	0.956	0.951
CTS	75	3199	3	3	0.961	0.961	0.961
SRS	231	3374	8	10	0.966	0.958	0.962

CB = Conveyor Belt; RT = Rotating Table; SR = Sliding Robot; PR = Production Robot; P&P S = Pick & Place Robot S Axis; P&P U = Pick & Place Robot U Axis; P&P L = Pick & Place Robot L Axis; CTS = Container Tray Slider; SRS = Sliding Robot Slider.

**Figure A1:**
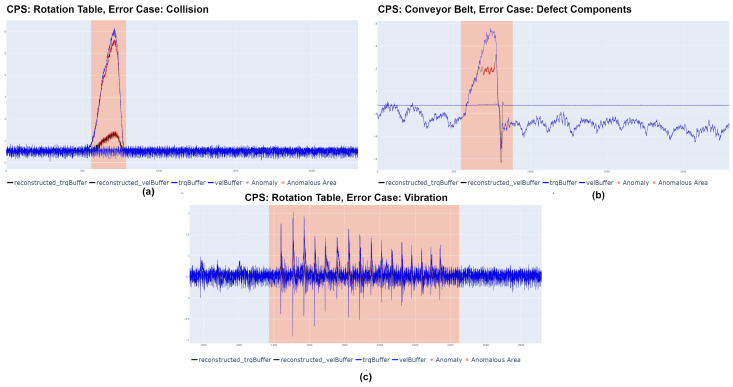
Additional Anomalous Samples.

## Data Availability

Limited accessibility to the dataset can be given in single cases.

## Appendix A

Table A1, Table A2 and Table A3 lists each individual item of Figure 4, Figure 5 and Figure 6 with a short description.

**Table A1:** Definition Table BPMN Diagram AD Installation Cycle Figure 4.

Item	Definition
Processing Unit Backend	The processing unit backend, an external device that can be removed after the installation process is finished, performs all heavy processing tasks in the AD Installation cycle to meet the previously explained industrial constraints of the CPPS.
Control Device	Unit which typically controls the industrial process.
Communication Interface	The interface of the embedded system to exchange data with the control device or the processing unit backend.
Embedded System	Part of the CPS which interacts with sensors and actors to monitor and control the mechanical system.
Mechanical System	Summarizes all mechanical components of the system.
Process data samples	Single packages of time series data from the individual CPS. Process data samples consist of features like position, torque, and speed sampled as time series data from the CPS.
Record Regular Process Data	Combined process data samples of all CPS collected from the normal process sampled over a defined time.
Collect Process Data Samples	Process data samples at a high sample rate are collected from the different CPS, combined, and sent to the control device as a data package.
Analysis	In the analysis, unnecessary features are automatically removed from the data, and important information like feature range and data types are collected.
Generate Configurations	Based on the analysis, configuration files are generated. The operator can manually tune this information, or the default values can be used.
AD Generation Cycle	Main cycle to create the preprocessor and train the model.
Deployment AD Pipelines	Each generated AD pipeline is exported and deployed to a separate CPS.
AD Production Cycle	Live integration and execution of the AD pipeline in the individual CPS.

**Table A2:** Definition Table BPMN Diagram AD Production Cycle Figure 6.

Item	Definition
In-memory data storage	A fast and effective data store that caches live data until it is passed to the AD pipeline for processing.
Record Live Process Data	Live process data is sampled at a high sample rate to an in-memory data storage to collect the needed data to execute the AD pipeline.
Execute AD Process Step	The collected live data is preprocessed and evaluated by the AD pipeline.
Deliver Results to Control Unit	The AD output can be delivered from the CPS to the control unit.
Notify Operator	Depending on the CPS, the Operator can be immediately notified by the separate CPS.
Shut Down AD	In this step, the whole AD production cycle can be switched off to free resources and stop the anomaly detection.

**Table A3:** Definition Table BPMN Diagram AD Generation Cycle Figure 5.

Item	Definition
Regular Process Data	Data collected from the normal process of the CPS over a defined time.
Preprocessed Data	Transformed and scaled regular process data by the chosen Preprocessor.
AD Pipeline	A combination of initialised Preprocessor and trained model.
Configuration	Contains necessary parameters for the separate steps of the generation cycle, e.g., the number of layers, filters per layer, loss function, and type of optimizer. Default parameters are automatically provided but can also be manually changed and tuned.
Preprocessing	In the preprocessing step, the regular process data is transformed by the chosen preprocessor. This scales the data provided, which normally consists of different ranges and units, to an equal numerical range.
Initialize Model	Here, the model is built based on the configuration. Therefore, the number of layers, filter, and type of each layer and the optimizer and loss function are set.
Train Model	In this step, the initialized model is trained with the preprocessed regular process data.
Evaluate Model	Depending on the evaluation method defined in the configuration step, the model is tested, the results are tracked, and the complete experiment is saved.
Optimize Model	In the optimization step, the hyperparameters are changed, influenced by the defined ranges and tuning parameters. The search algorithm declared in the configuration file searches over a generated search space for the best possible parameters.
Export AD Pipeline	Normally, after the tuning is finished, the AD pipeline is exported to the deployment step.

## Appendix B

To reach adequate prediction performance and fulfil the requirements defined in Section 3, several models were investigated. All models were tested and evaluated against a reduced test data set, consisting of the time series data of one CPS and a limited number of anomalies. No optimisations were made to the models. The test results can be seen in Table A4. Decisive characteristics for the choice of the model are the number of required parameters, the recognition rate and the time required for the training and the evaluation of the test data. The table clearly shows that shallow methods, despite their fast evaluation, have significant weaknesses in the recognition rate for dynamic and complex time series, as specified in [11]. The LSTMAE has a higher number of parameters compared to the other models. Due to the focused universal application and the limited process and memory resources, this is a major disadvantage. The CAE shows a slightly improved recognition in our test dataset compared to the AE architecture without convolutional layers. Based on the findings of [37,38,39,40,41,43], which identified the good performance of the ConvAE on industrial time series data, we have chosen the 1D-ConvAE as the model.

**Table A4:** Model Evaluation.

Model	Performance							Size	Avg. Time [ms]	
	**TP**	**FP**	**TN**	**FN**	**Recall**	**Precision**	**F1**	**Compelxity**	**Training**	**Evaluation**
OCSVM	400	25,005	19,633	138	0.7434	0.0157	0.0308	Low	37,041.6	36,811.1
iForest	467	13,709	30,865	71	0.8680	0.0329	0.0634	Low	1874.9	574.1
LSTMAE	8	1	693	1	0.8888	0.8888	0.8888	High	372,029.5	23,491.3
AE	8	2	692	1	0.8888	0.8	0.8421	Medium	13,151.2	18,720
1D-ConvAE	8	0	694	2	0.8	1	0.8888	Medium	113,227.6	20,045.2

OCSVM = One-Class Support Vector Machine [56]; iForest = Isolation Forest [57]; LSTMAE = Long Short-term Memory Autoencoder [58]; AE = Autoencoder [59]; 1D-ConvAE = One dimensional convolutional Autoencoders.

## Appendix C

Figure A1 shows additional showcases of anomalous samples. In Figure A1a the collision case is shown. As described in Section 6.2, the system detects typically heavy collisions. The production of light collisions is a difficult task that requires precise interference in the process. Therefore, a pole was prepared with a predetermined breaking point and applied against the rotation direction of the rotary table during regular operation. Figure A1b shows the case defect component. A container was manipulated to force pressure on the moving conveyor belt at a certain point. To create the error case seen in Figure A1c, minor shocks were manually applied to the plate of the rotation table. A specialist carried out all the different tests, considering safety aspects for the person and the system.
